# Transcatheter Tricuspid Regurgitation Repair—An Overview of Techniques and Eligible Patient Selection

**DOI:** 10.3390/jcm13226876

**Published:** 2024-11-15

**Authors:** Edme Roxana Mustafa, Daniela Marinescu, Cristina Florescu, Ionuț Donoiu, Octavian Istrătoaie

**Affiliations:** 1 Department 3, Medicine Faculty, Craiova University of Medicine and Pharmacy, 200349 Craiova, Romania; cristina.t.florescu@umfcv.ro (C.F.); ionut.donoiu@umfcv.ro (I.D.); octavian.istratoaie@umfcv.ro (O.I.); 2 Department 7, Medicine Faculty, Craiova University of Medicine and Pharmacy, 200349 Craiova, Romania; dtmarinescu@yahoo.com

**Keywords:** transcatheter repair, tricuspid valve regurgitation

## Abstract

Tricuspid regurgitation (TR) is frequently encountered in clinical practice. It is classified into primary TR (organic), which accounts for a minority of cases; and into secondary TR (functional), which has a higher prevalence. Although it can be asymptomatic for a long time, TR causes right ventricle dysfunction and increases hospitalizations for heart failure and mortality. In most patients with severe, isolated TR, surgery is not indicated due to the high surgical risk. In the last 10 years, transcatheter tricuspid valve repair became feasible with the following good results: reduction in TR severity, reverse remodeling of the right heart chambers, improvement in symptoms and reduction in hospitalization time. This paper presents the main transcatheter techniques and data from relevant trials that used these techniques, focusing on patient characteristics that define eligibility and high probability of repair. Information is provided regarding the observed benefits and the complications.

## 1. Introduction

Tricuspid regurgitation (TR) is frequently observed in clinical practice. The prevalence of moderate and severe TR is 0.55% in a community setting [1]. In the elderly, it is diagnosed in one in twenty-five individuals, with a higher frequency in women [1].

It is classified into primary TR (organic) which accounts for 8–10% of cases and secondary TR (functional), with a higher prevalence. The primary form occurs when there are intrinsic structural changes in the leaflets as in infective endocarditis, congenital disease, prolapse, rheumatic disease, carcinoid syndrome, etc. The secondary form occurs in conditions with right heart volume or pressure overload, in cardiomyopathies and in atrial fibrillation. In the secondary form, the tricuspid annulus dilatation and excessive tethering of the cusps are contributors to regurgitation. Secondary TR aggravates right ventricular (RV) dysfunction and represents a predictor of hospitalizations [2]. There is a direct correlation between TR severity and mortality. In patients with heart failure with reduced ejection fraction (HFrEF), which is the most common cause of secondary TR, the negative prognostic value of TR is maintained after multivariable adjustment including age, atrial fibrillation, left ventricle ejection fraction, E/e′ ratio, presence of pulmonary hypertension or right ventricular dysfunction [3]. In hospital, mortality after tricuspid valve repair or replacement varies from 8.1% to 10.9%, exceeding the corresponding values for the correction of mitral regurgitation or aortic stenosis [4,5].

Since 2015, various methods of transcatheter tricuspid valve repair (TTVR) have been successfully applied with significant improvement in patient clinical status and survival [6]. The number of patients is small compared to that in similar procedures performed for the mitral and aortic valves, but the achieved success is motivating. Transcatheter procedures cause fewer complications and are accompanied by fast recovery and shorter hospitalization time.

The following methods for TTVR are available: 1. tricuspid valve edge-to-edge repair (TEER); 2. tricuspid annuloplasty; 3. tricuspid valve orthotopic implant; 4. heterotopic valve implant in the caval veins.

This review focuses on a description of the TTVR techniques, on the indications and contraindications of TTVR, on the characteristics of eligible patients, on the short- and medium-term benefits attained and also on the periprocedural complications.

## 2. Inclusion and Exclusion Criteria in Trials Performing TTVR

TTVR is indicated in symptomatic patients with moderate or severe TR on optimal medical therapy, in which surgery is not an option because of the high risk. Most patients included in trials for TTVR had secondary TR but a small percentage also had primary disease, usually valve prolapse.

A good-quality image of the tricuspid valve on transthoracic (TTE) and transesophageal echocardiography (TEE) is necessary for guiding repair. Up to 20% of patients with TR can have a poor-quality image of the valve [6].

There are anatomical features of the tricuspid valve specific to each type of procedure which are used to predict the success of the repair (coaptation gap, effective regurgitant orifice area (EROA), tenting height and area, tricuspid annulus diameter, etc.). A large EROA > 1.5 cm^2^ or a coaptation defect >15 mm are contraindications for TEER [7].

Cusp calcification, valve cleft or perforation in the grasping area, other leaflet defects preventing device placement, active endocarditis, tricuspid stenosis, the presence of a biological prosthesis or a previous tricuspid valve repair are contraindications for TEER [6].

Other “specific” contraindications could be a tricuspid annulus circumference longer than Cardioband’s maximum length or the proximity of the annulus to the right coronary artery in cases of tricuspid annuloplasty or vena cava diameter larger than 34–35 mm and unsuitable vein morphology in cases of caval valve implants.

Common exclusion criteria are systolic pulmonary artery pressure sPAP above 60 mmHg, severe RV dysfunction—assessed as TAPSE (tricuspid annular plane systolic excursion) less than 13 mm, severe LV dysfunction, uncorrected severe mitral or aortic valve disease and a recent coronary syndrome or PCI within the prior 30 days [8]. The presence of a pacemaker or defibrillator lead is not a contraindication per se but it may become so if the lead impinges on a cusp, severely restricting its motion. A central localization of the lead instead of a commissural one can make TEER difficult. Pacemaker implantation in the previous month is a temporary contraindication.

Extracardiac pathologies that reduce life expectancy to less than 1 year, as well as bleeding disorders or hypercoagulable disorders, are contraindications [9,10]. Deep vein thrombosis affecting the veins used for implantation and intracardiac thrombi are also contraindications. A contraindication to DAPT and anticoagulant therapy precludes repair [8]. Some chronic diseases like chronic dialysis, uncorrected anemia (Hb ≤ 9 g/dL) and cardiac cachexia were exclusion criteria in some studies [10].

## 3. The Profile of Patients Included in TTVR Trials

The patients with TR included in transcatheter repair studies are complex and frail. An analysis of the patient characteristics from the TriValve Registry (the largest database of patients treated with TTVR) is relevant. The registry included 249 patients with severe TR. The patient mean age was 77 years old, with a predominance of women and with an average EuroScore II of 6.4% [11].

The most common diseases were the following: heart failure with reduced ejection fraction (in 25% of cases), mitral or aortic valvular disease (active or already treated in 68% of patients), atrial fibrillation (in two-thirds of cases). Chronic obstructive pulmonary disease was diagnosed in 25% of cases. Previous repair of the mitral or aortic valve (either by surgery or by transcatheter) was encountered in 10.8% and 11.6% of cases. Twenty percent of patients had an old myocardial infarction. A third of cases had a pacemaker or an ICD inserted. The mean left ventricle ejection fraction (LVEF) was 49 ±14% and the average systolic pulmonary artery pressure was 43.6 ± 16 mmHg [11]. Other common pathologies were diabetes mellitus in a third of cases, cerebrovascular disease and chronic kidney disease [11].

## 4. Description of Individual TTVR Techniques, Profile of Eligible Patients and Results from Relevant Trials

### 4.1. Transcatheter Tricuspid Valve Edge-to-Edge Repair (TEER)

**Figure 1:**
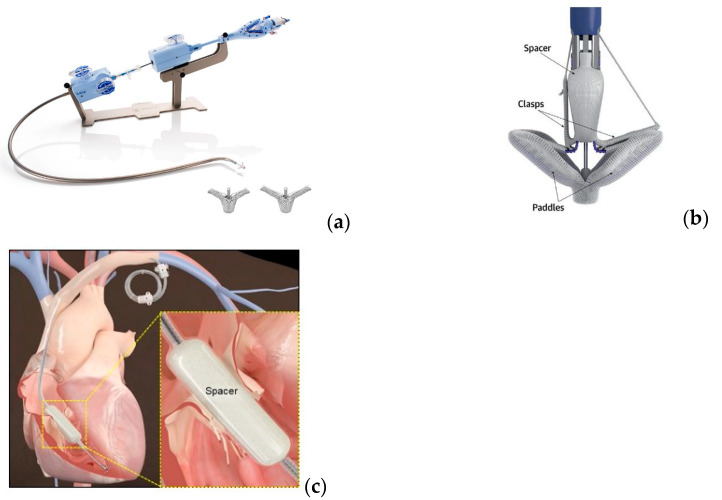
(**a**) TriClip-delivery system and clips, Abbott [12]; (**b**) Pascal device, Edwards Lifesciences [13]; (**c**) Forma device, Edwards Lifesciences [14].

TEER, a technique which improves cusp coaptation, was first performed using MitraClip and later using dedicated devices, the TriClip and Pascal devices. The devices contain a steerable guide catheter (SGC), clip delivery system (CDS) and the clips. The clip’s arms can independently grasp each leaflet. MitraClip and TriClip (Abbott, Hongkong, China) have four clip sizes which allow attachment to a leaflet length that ranges from 6 to 12 mm. TriClip has some adjustments that allow greater mobility and multidimensional steering. The Pascal device (Edwards Lifesciences, Irvine, CA, USA) resembles TriClip and has a central spacer that reduces the coaptation gap. These devices and the FORMA device are shown in Figure 1.

Multiplane 2D TEE and real time 3D TEE and fluoroscopy are necessary to guide the implantation. Intracardiac echocardiography is also used. Devices are commonly inserted through the femoral vein, by navigating through the inferior vena cava and right heart. The clip is carefully positioned below the tricuspid leaflets, avoiding entrapment in the chordae tendinae and reaching for the largest coaptation gap. The clip is placed perpendicular to the coaptation line. The implantation requires general anesthesia. Double antiplatelet therapy (DAPT) is prescribed for at least 1 month after implantation to prevent thrombosis.

Relevant information about TR parameters used in selecting patients for TEER repair comes from the TriValve Registry. In this registry, MitraClip was used for TV repair. A total of 97% of the cases had severe TR and the majority (95.6%) were in heart failure classes III and IV NYHA. The mean value of TR-EROA was 0.7 ± 0.53 cm^2^, the vena contracta was 9.9 ± 4.1 mm, the TR coaptation gap was 5.5 ± 3.3 mm and the mean value of the tenting area was 2.3 ± 1.5 cm^2^. Approximately one to five clips were implanted for TR correction; in most cases, two clips were needed (43.8%). TEER led to a reduction of at least one grade in TR severity in 89.2% of cases and 77% of cases had mild or moderate TR at 30 days follow-up. In 69% of the patients, the signs of heart failure improved, the patients being in NYHA classes I and II postprocedural. TEER did not lead to a significant increase in the tricuspid gradient [11].

The authors of this registry consider that the predictive factors of TEER failure are a coaptation gap ≥ 6.4 mm, an EROA ≥ 0.7 cm^2^, a tenting area ≥ 3.2 cm^2^ and the absence of a central jet or the jet’s origin being in a different location than the anteroseptal commissure [11]. In this registry, mortality at 30 days was 2.8% and at 1 year reached 20%, while mortality and unplanned hospitalizations for HF at 1 year were 35% [11].

In C. Besler’s study, in the univariate analysis, the values that predict successful repair were similar: EROA < 0.6 cm^2^, vena contracta < 11 mm, tenting area < 2.1 cm^2^. In this study, the strongest predictor of procedural success was a coaptation gap value below 7.2 mm. At coaptation gap values above 10 mm, the success of the repair was obtained in less than 30% of cases [7].

Another reference study is TRILUMINATE which included 85 patients with severe symptomatic TR in whom the TriClip device was used for repair. The TR severity parameters were an EROA of 0.65 ± 0.03 cm^2^ and a vena contracta of 17.3 ± 0.7 mm (higher than in the TriValve Registry). TEER led to a decrease in EROA to 0.32 ± 0.05 cm^2^ and in the vena contracta to 0.78 ± 0.05 mm, representing a reduction of 50, respectively, 54% [9]. RT severity was reduced by one grade in 87% of cases. After 30 days, 63% of cases had mild or moderate TR and 83% of cases were in NYHA classes I and II.

Comparing TR severity after 30 days and 1 year, the authors found a further reduction in severity in 35% of cases, no change in 44% and a one-grade increase in severity in 21% of cases. The torrential or massive forms of TR are less likely than the severe form to reduce to moderate TR after repair. However, even in these extreme forms of TR, a reduction in severity by one grade can be achieved [9].

Mortality after 1 year was 7.1% and hospitalizations decreased by 40% [9].

In two studies, investigators included patients with a larger TR vena contracta. In Meijerink’s study, the average value was 14 mm; and in Ruf’s study, the value was 15.5 mm [15,16]. A reduction in RT severity by one grade was attained in 81%, respectively, 98% of cases, but mild or moderate TR was ultimately obtained in fewer patients (57%, 54%, respectively). In Meijerink’s study, for coaptation gap sizes lower than 10 mm, procedural success was obtained in 93% of cases, while for values above 10 mm, the success rate was reduced by half (43%). The largest coaptation gap at which the procedure was applied was 13 mm [16]. According to Ruf, a gap coaptation size ≤ 8.4 mm is a predictor of obtaining a moderate or mild TR after repair. In Ruf’s study, when TEER was performed in patients with gap coaptation sizes above 10 mm, heart failure improvement with one NYHA class was obtained, but 6MWD did not improve [15].

Additional information about RT severity parameters and the results obtained by TEER from 12 reference studies, on a total of 848 patients, are shown in the Supplemental Tables S1 and S2.

In the CLASP-TR study, TEER reduced the severity of TR by one grade in 85% of cases, by two grades in 70% and by three grades in 26% of patients [17]. The results were similar after 30 days. TR severity reduction is accompanied by an improvement in the NYHA class. This improvement varies in studies depending on the patient characteristics. In Nickenig’s trial, 37% of patients were in NYHA classes I and II at the end of the follow-up period, while in Sugiura’s trial, 93% of patients were in the same NYHA classes [18,19].

The following parameters can predict a successful tricuspid valve edge-to-edge repair:

- Coaptation gap < 7(10) mm;
- Tethering height ≤ 10 mm;
- Mobile leaflet length > 7(8) mm;
- Mean tricuspid valve gradient ≤ 3(5) mmHg;
- TR jet origin located centrally or at the anteroseptal commissure [6].

In most cases, two or more clips are necessary to reduce TR. The best results are obtained by placing the clips at the level of the anteroseptal commissure or at both anteroseptal and posteroseptal commissures [11].

After placement of clips, the tricuspid valve opening can show three orifices (triple orifice technique—TOT) or a single orifice (bicuspid appearance). The reduction in TR severity is similar. In the case of TOT, the diameter of the tricuspid ring is reduced more, hence resulting in a possible benefit over time in maintaining the TR reduction [20].

The TEER procedure does not cause a significant increase in the diastolic pressure gradient at the tricuspid level [11,13,21]. In the TRILUMINATE study, 4.7% of patients had a mean gradient greater than 5 mmHg at 1 year follow-up, all patients being asymptomatic, with no necessary reintervention [9].

Simultaneous transcatheter repair of the mitral and tricuspid valves is feasible and shows the best reduction in TR severity and in mortality compared with single valve repair. This situation was encountered in 32% of patients in the TriValve Registry [22].

The PASCAL and MitraClipXTR devices proved to be equally effective for the treatment of TR both in terms of reducing TR severity and improving NYHA class, 6MWD or mortality [19].

The reduction in TR severity is accompanied by RV reverse remodeling. M. Orban et al. documented the magnitude of this process at 6 months after TEER. The RV end diastolic area decreased by a mean value of 12.3%, the RV end diastolic volume by 16% and the RV end systolic volume by 6.8%; and the septolateral diameter of the tricuspid ring decreased by 6%. While the TAPSE and RV-FAC (right ventricle fractional area change) did not change, the RV-EF decreased by 5.8%. The EF reduction was due to the decrease in preload and not to the worsening of RV function. The TAPSE/PAPsystolic ratio, a parameter that reflects the RV–pulmonary artery coupling, improved from 0.36 preinterventional to 0.42 postinterventional. Vena contracta reduction was a predictor of RV reverse remodeling [23].

Most of the right heart remodeling takes place in the first 30 days, but the process can continue for up to 1 year [9].

In TEER studies, mortality after 30 days varied largely between trials. Some studies had no mortality after 30 days [15,17,20] while others reported 10% mortality [16]. Mortality after 1 year varied between 7.1% and 37.5%, reflecting the complex pathology and the advanced stage of heart failure in some patients [9,20]. In C. Besler’s study, successful RT repair led to a significant decrease in mortality (15% versus 46% in cases of procedure failure) and a reduction in HF hospitalizations (11% versus 50%) over a follow-up period of 184 days [7]. The simultaneous repair of the mitral and tricuspid valves reduced mortality two times more than isolated mitral valve repair [11].

The clinical outcomes after TEER are strongly influenced by the cause of TR. Patients with TR secondary to mitral regurgitation (MR) or atrial fibrillation have a lower risk of death, heart failure hospitalization or reintervention compared to patients with TR secondary to dialysis or pulmonary arterial hypertension (PH). Dialysis patients have the highest mortality 1 year after TTVR (33% at 1 year). Patients with PH have the highest rate of death or hospitalization for HF or reintervention [24].

An analysis of the outcomes after TEER in the different forms of HF showed similar procedural success (86% versus 78%), improvement in NYHA class in both forms of HF and a larger reduction in mortality and hospitalizations in heart failure with preserved EF(HFpEF) compared to heart failure with reduced EF (HFrEF)-30% versus 50% [25].

The main complications described in TEER studies are bleeding, stroke, myocardial infarction, acute kidney injury, infections, cardiac tamponade, reversible low cardiac output syndrome (in conditions of increased RV afterload and transient RV dysfunction), arrhythmias. These complications are similar to those encountered in cardiac surgery, but occur less frequently. Single leaflet device attachment (SLDA) occurred in 1–10% of cases; in most cases it did not cause a worsening of TR and conversion to surgery was not necessary [16,17]. Only in a few cases of procedure failure was surgery or a new transcatheter intervention necessary.

These complications are presented in the Supplemental Table S3.

### 4.2. TTVR Using the FORMA Device

FORMA (Edwards Lifesciences) is a special device that reduces the regurgitation orifice without traction of the cusps. FORMA contains a spacer (foam-filled polymer balloon), round and tubular shaped. The spacer is inserted through a rail that is fixed at one end in the subclavian vein and at the other end in the right ventricle. The fixation in the right ventricle is made by a nitinol anchor with curved prongs that attach to the myocardium in the lateral wall of the RV near the apex. The venous access is at the subclavian vein by surgical cut-down or percutaneous approach.

The spacer is placed in the central part of the tricuspid valve, reducing the regurgitant orifice and creating a new surface for leaflet coaptation. For proper positioning, fluoroscopy and TEE are mandatory. The operator chooses the best position that significantly reduces TR.

The FORMA device has a standard length of 42 mm and its width varies from 12 to 15 to 18 mm. FORMA requires anticoagulation or DAPT in the first 3–6 months after implantation to prevent device thrombosis [26].

A literature search identified two studies that used the FORMA device for TR repair. The parameters of TR severity are similar to those from TEER studies: the average EROA varied between 0.92–1.1 cm^2^ and the average vena contracta varied between 11.8–16 mm, with most patients having severe TR. In more than 50% of the patients, TR severity was reduced to mild or moderate at 30 days and the NYHA class improved in 66% of the cases. TR correction led to RV reverse modeling. Mortality was 6.89% at 30 days and 29% at 32 months [21,26]. The FORMA implant is more laborious than the TEER technique. The risk of RV perforation varied between 5% and 11%. Other complications are similar to those in TEER. Information about possible complications is presented in the Supplemental Table S3.

### 4.3. Transcatheter Tricuspid Annuloplasty

Transcatheter tricuspid annuloplasty is a procedure that shortens the tricuspid ring. It is indicated in symptomatic patients with severe secondary RT, with high surgical risk, in which the valve anatomy is not suitable for TEER. The ideal profile is atrial TR in which there is a dilation of the annulus without significant cusp tethering and a sufficient distance between the right coronary artery and the annulus. Three devices, Cardioband, TriAlign and TriCinch, were used in clinical trials, the largest experience being with Cardioband and this one is the only device still in use.

The Cardioband device (Edwards Lifesciences) is an arch-shaped polyester tube in which a metal wire is inserted; the tube is attached to the tricuspid ring with the use of anchors. The insertion on the ring should be on the atrial side, above the cusps’ hinge point. Implantation starts at the level of the anteroseptal commissure and it continues along the anterior and posterior parts of the tricuspid ring, requiring a maximum of 17 anchors for good fixation [10,27]. The device is inserted by transfemoral venous puncture, through a 26 F sheath.

**Figure 2:**
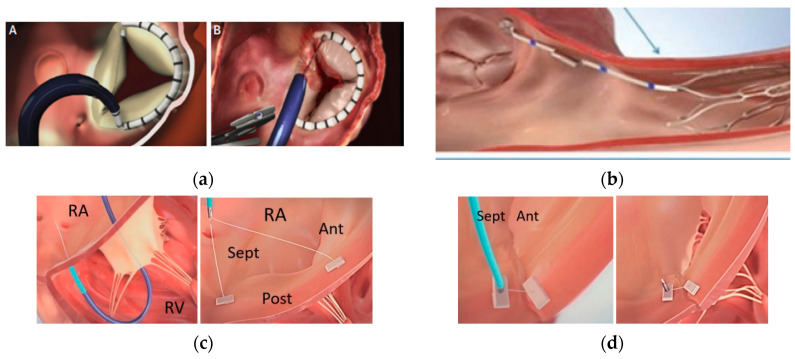
(**a**) Cardioband (Edwards Lifesciences) implantation [10]; (**A**) Fixation of the Cardioband on the tricupsid ring; (**B**) Shortening of the metallic wire, of the tricuspid ring and reduction of tricuspid regurgitation orifice; (**b**) TriCinch (4Techcardio Ltd., Ankara, Turkey) implantation [28]; (**c**,**d**) TriAlign (Mitralign Inc., Tewksbury, MA, USA) implantation [29].

It is necessary to place a visible wire in the right coronary artery (RCA) to ensure the safety of placing the anchors, without coronary perforation. After fastening, the device allows for the rotation and shortening of the metal wire and the shortening of the tricuspid ring. The procedure is performed under TEE and fluoroscopic guidance. Intracardiac echocardiography is useful in guiding the fixation in the lateral portion of the tricuspid ring. Before the intervention, TTE, 3D TEE and cardiac CT scan are necessary to measure the dimensions of the tricuspid ring. The Cardioband and other similar devices are shown in Figure 2.

Four implant sizes are used, corresponding to tricuspid ring circumferences in diastole of 89–96 mm, 97–104 mm, 105–112 mm, 113–120 mm. The specific contraindications for Cardioband insertion are severe tethering of cusps over 10–15 mm, eccentric RT jet (pseudo flail), a ring too wide for the device, proximity of the area of device insertion and RCA, thin annular anatomy, thick leaflets, proximity of pacemaker/ICD lead to the cusps [10].

After the Cardioband implant, antiplatelet treatment with aspirin is required for 30 days [27].

Three studies including a total of 121 patients used Cardioband for the correction of severe TR. Tricuspid annuloplasty reduced the diameter of the tricuspid ring by more than 10%, which led to a 35% decrease in TR-EROA and to a reduction of one grade in TR severity in more than 50% of cases. Before repair, over two-thirds of patients had severe TR, and annuloplasty reduced the severity to mild or moderate in more than 44% of patients. NYHA class improved significantly. Mortality after 30 days ranged between 0 and 6.7% and reached 10% after 1 year [10,27].

The following complications were observed: cardiac tamponade, coronary complications (RCA perforation, aggravation of pre-existing lesions or occlusion of branches), atrioventricular block, ventricular arrhythmias. Some coronary complications required PTCA and stenting. Information about complications is presented in the Supplemental Table S3.

TriAlign (Mitralign Inc.) is a device which brings closer the posteroseptal (PS) and anteroposterior (AP) commissures of the tricuspid valve with the folding of the posterior cusp. The valve acquires a bicuspid aspect. The technique consists of passing a wire through the tissue of the tricuspid annulus at the level of the PS commissure; the wire is snared in the RA and a pledget is placed. A second wire is passed through the annulus at the AP commissure at 2.4–2.8 cm from the first wire. Then, folding is performed and secured with a lock device [29].

TriAlign was used for the correction of moderate and severe TR in 15 patients. Technical success (defined as correct implantation of the device) was achieved in 80% of cases at 30 days. TriAlign reduced TR-EROA by 37% and the diameter of the tricuspid ring by 2.4%. No data are available on the percentage of patients with successful TR reduction. NYHA class decreased significantly. The following complications occurred: coronary complications (which required PTCA of RCA) and single pledget detachment which did not aggravate TR and did not require intervention or conversion to surgery [29].

TriCinch (4Techcardio Ltd.) is a device that contains a corkscrew anchor, a self-expanding nitinol stent and a Dacron band which connects the two. The anchor should be fixed to the tricuspid annulus at the level of the AP commissure, while the stent is fixed in the inferior vena cava. The Dacron band pulls the anchor towards the inferior vena cava, causing plicaturation of the posterior cusp and improving cusp coaptation [28]. The amount of tension applied is varied until the best reduction in TR is achieved. Later, the device’s anchoring site changed from the endocardium to the pericardial space, to increase stability. A first trial with 24 patients reported procedural success in 81% of cases, TR severity reduction by one grade in 91% of cases, no in-hospital mortality and pericardial effusion in 8.33% of cases. At 6 months follow-up, anchor detachment was diagnosed in five patients (20%). TriCinch and TriAlign are no longer available for use.

### 4.4. Transcatheter Replacement of TV (TRTV) or Orthotopic Transcatheter Tricuspid Valve

This is the preferred option in patients with severe TR, with high surgical risk, in which the anatomy of the TV (a large coaptation gap, non-central jet, calcification, valve immobility or severe tethering) makes TEER unfeasible. Parameters such as the diameter of the tricuspid ring, the size of the right heart chambers, the diameter of the inferior vena cava (IVC), the distance and the angulation between the IVC and the tricuspid ring plane are obtained by CT scan. They are necessary to establish feasibility and select the valve size. The implantation of the valve is performed by transfemoral approach; the positioning is performed under fluoroscopic and TEE control. Complications are related to the difficulty of anchoring the prosthesis to the flexible tricuspid ring. Suboptimal fixation leads to regurgitations (intravalvular and paravalvular) and increases the risk of embolization and thrombosis [29]. The compression exerted by the different components of the valve (frame, anchors) on the interventricular septum and the His bundle can cause complete atrioventricular block and increase the need for pacing. Valves require oral anticoagulation in the first 6 months, either with vitamin K antagonists or with NOACS. More than half of patients in trials received both anticoagulants and aspirin. After 6 months, the physician can maintain anticoagulation or switch to DAPT [30].

There are several valve models, most of them being implanted in just a few patients. The greatest clinical experience is with the Evoque valve.

The Evoque valve (Edwards Lifesciences) has a self-expanding nitinol frame with a bovine pericardial valve.

**Figure 3:**
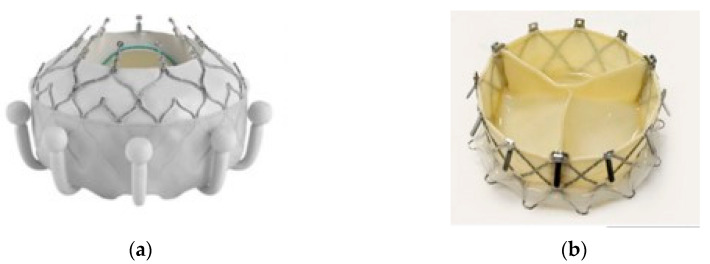
(**a**) Evoque valve (Edwards Lifesciences); (**b**) Gate System/Navigate valve (NaviGate Cardiac Structures Inc., Lake Forest, CA, USA) [30].

It has an intra-annular skirt to prevent leakage. The first stage of implantation consists of prosthesis fixation on the tricuspid ring in the upper part corresponding to the native cusp hinge point. The prosthesis is available in 44, 48 and 52 mm sizes. When choosing prosthesis diameter, the degree of oversize in relation to the tricuspid ring diameter in diastole should be small, due to the risk of rupture. In the reference study, the degree of oversize was 3 ± 4.1%. After fixing on the tricuspid ring, nine U-shaped anchors are exposed on the ventricular surface of the tricuspid cusps, improving fixation. Finally, the atrial portion of the prosthesis with a sealing skirt is released. The positioning is performed using fluoroscopy and TEE [31]. The device is inserted through a 28F sheath. The Evoque valve is shown in Figure 3.

The first clinical trial with the Evoque valve included 25 patients. The technical success was achieved in 92% of patients, without in-hospital mortality or conversion to surgery. The mean diameter of the native tricuspid ring was 45.2 ± 2.7 mm. The optimal angle between the IVC opening and the tricuspid ring was 83^0^, and the distance between the IVC opening and the tricuspid ring was 5.8 ± 1.8 mm. After Evoque implantation, TR severity significantly decreased to moderate or less in 90% of cases and the symptoms improved to NYHA class I or II in two-thirds of patients at 30 days follow-up. All patients survived after 30 days. Subsequent to prosthesis implantation, residual regurgitation was more often intravalvular rather than paravalvular [31]. TRTV is feasible in patients with pacemaker leads. Among the complications encountered, we have mal positioning of the valve (too low in the ventricle, below the plane of the ring) which led to severe central TR and was treated with the implantation of a SAPIEN valve-in-valve. Other complications were valve thrombosis or complete atrioventricular block [31].

TRISCEND is the reference study that used the Evoque valve. This study included 176 patients, 88% of them with severe, massive or torrential TR and with a predominance of the secondary TR form. A total of 75% of the patients were in NYHA classes III and IV. TR severity reduced to mild or less in 97.65% of cases and the result was maintained after 1 year. After TTVR, mild and moderate paravalvular regurgitation occurred in 10.6% and 1.2% of cases. TR correction was accompanied by right heart reverse remodeling, by an increase in the cardiac output, by an improvement in heart failure symptoms and by an increase in the exercise capacity and in the quality of life. After 30 days, the cardiovascular mortality was 1.7% and non-elective re-intervention on the tricuspid valve was required in 2.3% of cases. After 1 year, the mortality was 9.1% and hospitalizations for HF were 10.2%, with a 75% reduction compared to the baseline. Mortality after 1 year and hospitalizations were the lowest reported for transcatheter replacement therapies, a result that correlated with the successful TR correction. In 5.6% of cases, there were unsuccessful device implants due to insufficient imaging or unfavorable anatomy. The patients in this study will be monitored for up to 5 years, providing useful data on the durability of the RT correction [32].

Another available valve is the Gate System/Navigate transcatheter heart valve (NaviGate Cardiac Structures Inc.). This is a nitinol self-expanding stent housing a trileaflet equine pericardial valve. It has an anchoring system with 12 tines on the ventricular side and 12 atrial winglets. It needs a 42Fr sheath for insertion which is performed by minimally invasive right thoracotomy [30].

There are four sizes available corresponding to diameters of the tricuspid ring between 40 and 52 mm. CT scan examination is mandatory to measure the diameters and areas of the tricuspid ring and to find the best intercostal space which allows for a perpendicular access to the tricuspid valve.

The fourth intercostal space on the middle or posterior axillary line is usually chosen. The right lung is deflated, the pericardium is opened and incision is performed in the middle part of the right atrium (RA) anterior wall. The valve diameter should oversize by 2–5% the tricuspid ring [33].

The Gate System was implanted in five patients with severe TR. Technical success was obtained in all cases, without periprocedural mortality. TR was significantly reduced, with 80% of cases being mild and with 20% being mild–moderate, while the symptoms improved. The complications encountered were AV block, bleeding, thrombosis of the prosthesis, fistula between the RV and the noncoronary Valsalva sinus [33]. Further information on studies with transcatheter valves is presented in the Supplemental Tables S1–S3.

### 4.5. The Heterotopic Caval Veins Valve Implant (CAVI)

This prevents the blood outflow from the RA into the venous system. CAVI is indicated for patients with severe symptomatic TR, in NYHA classes III and IV, with high surgical risk and large tricuspid EROA or severe annulus dilatation that overcome the possibility of repair by other transcatheter techniques. The first implants used a non-dedicated device, the Edwards Sapien valve, followed by the possibility of using two devices specifically designed for the venous system: TricValve and Tricento. CT scanning is necessary before the intervention, for describing the anatomy of the caval veins. The presence of various caval abnormalities, like congenital abnormalities, aneurysms and stenosis, and the presence of large venous branches opening close to the usual implantation region contraindicate CAVI. A significant caval reflux (V wave amplitude > 25 mmHg at catheterization) is needed. The presence of pacemaker leads is not a contraindication [34]. CAVI is performed under fluoroscopic guidance. TTE is necessary during follow-up.

The first CAVI procedures consisted of stent implantation in both caval veins or only in the inferior vena cava (IVC), close to the junction with RA; and in the second step, balloon expandable valves (BEV) like Edwards Sapien/SapienXT or DirectFlow Medical were fixed in the stented parts. For BEV, the IVC diameter at the diaphragm and the superior vena cava (SVC) diameter at the RA junction should be less than 30 mm, in order to avoid valve migration or embolization [34]. The CAVI procedure is shorter compared to other transcatheter repair methods and the same is true for the learning curve for implantation [34].

The TricValve system is made up of two self-expanding nitinol prostheses, each containing a bovine pericardium valve. They are inserted through the femoral vein approach, using a 27.5F delivery catheter. The prosthesis for SVC has a dilated central part (belly) which houses the valve. The inner part of the prosthesis is lined with a polytetrafluorethylene skirt to prevent paravalvular leakage. The belly should be positioned immediately above the crossing of the SVC and the right pulmonary artery, and the distal end of the prosthesis should be near the drainage of the brachiocephalic trunk. A minimum length of 5 cm is required between the brachiocephalic trunk and the cavoatrial junction. A shorter SVC, with proximal tapering, increases the risk of prosthesis migration [34,35].

**Figure 4:**
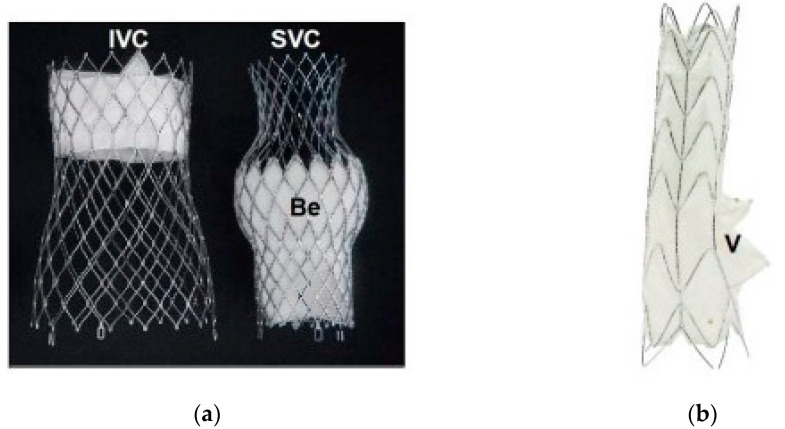
(**a**) TricValve; (**b**) Tricento device [34].

The proximal part of the IVC prosthesis should penetrate 5–12 mm into the RA. The first 20 mm have a skirt and contain the valve. The skirt reduces the paravalvular leaks. The covered portion is short so as not to occlude hepatic vein drainage. A distance of at least 5–15 mm between the hepatic vein drainage and the RA is recommended. The prosthesis has high radial force in the area containing the valve to fix to the vein wall and to reduce the risk of valve deformation. The other parts of the prosthesis have low radial force and a large diameter to facilitate fixation without traumatizing the vein wall. When choosing the size of the prostheses, it is necessary to oversize by 10–40% (in the belly part for the SVC and in the proximal part for the IVC prosthesis) compared to the vein size at CT scan. Initially, TricValve could only be used for vena cava diameters below 35 mm; later, larger prostheses became available [35]. TricValve and similar valves are shown in Figure 4.

In two studies, 60 patients with severe TR were treated with CAVI using Sapien Valve or TricValve. The procedural success, defined as the optimal fixation of the prosthesis and a reduction in backflow and pressure in the caval veins, was obtained in over 92% of patients [32]. The pressure in the IVC was reduced by 5–10 mmHg [35]. Congestion diminished in 72.3% of cases, and complete resolution of congestion was obtained in 58.6% of cases [36].

At 6 months after CAVI, the quality of life and the NYHA class significantly improved [34]. Hospital mortality after CAVI ranged from 2.8 to 16% and in most cases it was not related to the procedure. At 6 months after CAVI in patients with severe TR, the mortality was 8.5% and the heart failure hospitalization rate was 20%, values similar to other TTVR methods [36]. The longest follow-up of a patient after CAVI was 51 months and it showed normal functioning valves [35].

CAVI patients need anticoagulation with vitamin K antagonists (VKA) or NOACS at least in the first 3 months. In the reference studies, due to a high percentage of patients having permanent atrial fibrillation, anticoagulant therapy was continued for the long term.

The following complications occurred: valve migration (more common for Sapien valves), leaks, transient shoulder pain (produced by the compression of sensitive branches of the phrenic nerve close to the IVC prosthesis), thrombocytopenia (possibly due to a reaction to the pericardium leaflets), atrioventricular block and the need for cardiac pacing, prosthesis thrombosis despite anticoagulation. Surgery was required in a few cases for valve migration in the RA [35,36].

Tricento is a self-expandable nitinol stent graft spanning from the IVC to the SVC and crossing the RA. The nitinol stent is covered by thin porcine pericardium on an average length of 13.5 cm. It contains a bicuspid valve placed on the lateral wall of the stent graft which allows venous blood drainage into the RA and prevents systolic backflow. The leaflets are made of porcine pericardium. The device anchoring is obtained by oversizing the stent elements in the area of overlap of the stent and caval wall. The device is custom-made according to the patient anatomy, so there are no contraindications related to caval vein diameters. The device is introduced through the common femoral vein using a 24F sheath. The implantation is fluoroscopy-guided, while radiopaque markers facilitate the orientation of the graft. TEE can be used to evaluate the intra-atrial part and the valve. Postprocedural anticoagulation with VKA or NOACS is required [34].

The Tricento device was implanted in 21 patients with severe TR and high surgical risk. The patients were severely symptomatic, 95% being in NYHA classes III and IV and with frequent hospitalizations for heart failure [37].

The implantation was successful in 100% of cases. There were no cases of in-hospital mortality. After 2 months, symptoms significantly improved and 65% of the patients were in NYHA classes I or II. Mortality after 1 year was 24% and the heart failure hospitalization rate was 19%. The following complications occurred: paraprosthetic leakage, transient systemic inflammatory syndrome (without bacteremia), asymptomatic strut fracture of the intra-atrial part of the device. The fractures did not affect the competence of the valve. They correlated with high systolic pressures in the RA over 25 mmHg [35]. Further information on studies that used CAVI is presented in Tables S1–S3.

CAVI improves heart failure symptoms and quality of life, but does not reduce TR severity and does not improve RV function or cause minor reduction in RV volumes in the follow-up. As for other transcatheter procedures, the durability of the repair and the long-term benefits are not known.

M. Taramasso et al. analyzed the characteristics of patients with TR that underwent various TTVR methods, using data from the TriValve Registry. Most patients were treated with MitraClip (55%), followed by Trialign (16%), TriCinch (14%), FORMA (7%), the Cardioband device (5%) and CAVI (3%). Patients’ characteristics including parameters of TR severity were similar in patients treated with the various repair methods. Only for CAVI patients were the tricuspid annulus and vena cava significantly larger than for other methods. The procedural success and improvement in NYHA class were similar. The number of patients with moderate or less TR severity after TTVR was higher in patients that underwent concomitant mitral and tricuspid valve repair [22].

## 5. Discussion

TTVR is an efficient method of correcting severe TR in patients at increased surgical risk. Reduction in TR severity leads to RV reverse remodeling, improves symptoms and reduces hospitalizations for heart failure and mortality. TTVR in patients with HF is an accessible option that substantially improves survival compared to guideline-directed medical therapy (GDMT).

In Wang’s study, survival at 20 months after TTVR was 75.8% compared to 48.4% in the case of patients with TR treated only with GDMT. TTVR increases freedom from mortality and hospitalizations for heart failure (61.5% versus 45.9%). In this study, patients with TTVR presented more severe forms of TR, with more dilated and dysfunctional RV compared to patients treated with GDMT [38].

TTVR can successfully replace surgery in secondary TR and in some forms of primary TR.

Compared to surgery, TTVR is associated with lower in-hospital mortality, fewer complications, shorter hospital stays and lower care costs [39]. It should be mentioned that the favorable results of TTVR were obtained in reference centers, with experience in percutaneous techniques and with a large number of treated patients.

It must be taken into account that there is a learning curve for each technique and there may be failed procedures that lead to open chest surgery or death. A good selection of patients, including those with valve anatomy, clinical characteristics, operative risk and possible complications, together with knowledge of the repair techniques, is required for a favorable result.

TRI SCORE is a new risk score for in-hospital mortality prediction in patients requiring isolated tricuspid valve surgery. It includes eight parameters among which there are clinical, biological and imaging variables. It is a more accurate predictor of in-hospital mortality risk than EUROSCORE and EUROSCORE II. It also predicts the risk of major complications and mortality after 1 year. TRI SCORE could be used for selecting the optimal repair strategy, with a high score steering the decision towards transcatheter options [40].

In order to choose the best method for TTVR, multimodal imaging of the heart and vessels is needed together with analysis of the biological state and comorbidities as well as discussion of each case with the experts.

The above-mentioned studies described the anatomical characteristics and severity parameters of TR considered optimal for a successful repair for each technique. On the other hand, some patients with less favorable valve anatomy were also included in the studies and even in their cases, positive results were obtained—for example, a reduction in the severity of torrential TR by one or two grades—which led to an improvement in symptoms.

There are four methods of TTVR. TEER and annuloplasty are the most used repair methods; they preserve the valve anatomy and improve coaptation dynamics. They can be considered physiological correction methods. In theory, TEER is the best option for the ‘’ventricular’’ type of TR where the coaptation gap is not very large (ideally, below 8 mm), with some degree of tethering and without excessive dilation of the tricuspid ring [39].

Annuloplasty is the best option for the “atrial” type of TR with significant dilation of the tricuspid ring but without excessive tethering (below 8–10 mm) of the cusps and with a sufficient distance of the right coronary artery from the device attachment point (hinge point) [41].

Contrary to these premises, in older studies, the TR severity parameters (coaptation gap, EROA, tethering distance, tricuspid ring diameter) in patients who benefited from TTVR did not differ significantly between these two methods. Two recent studies comparing the two repair methods showed that more patients with torrential RT can be treated by AP compared to TEER [41,42]. AP is a more laborious procedure with a significantly longer duration than TEER. The combination of the two methods (AP and TEER) in the case of persistent severe regurgitation can be used in some patients.

The other two methods—the transcatheter replacement of the TV and CAVI—are less “physiological” methods of TR correction, being intended for advanced forms of the disease, with severe changes in the valvular anatomy where TEER and AP cannot be used.

Transcatheter TV replacement is a laborious method, with a long implantation time and a higher risk of complications related to the large size of the prosthesis. TR severity reduction is accompanied by reverse RV remodeling. CAVI requires a technique that is easier to apply and has a shorter implantation time. CAVI improves heart failure symptoms and quality of life, but does not reduce TR severity and does not improve RV function. Long-term benefits seem less likely than with the other methods.

Our study presents limitations related to the selection of the relevant studies from the (large) volume of existing papers in the literature. There is also a tendency in the literature to publish more information about new methods like the percutaneous techniques and to publish more successful results than less favorable ones.

Future trends: An early diagnosis of the disease, when there is less remodeling of the tricuspid valve and of the right heart, would allow for the use of TTVR in more patients. Also, TTVR could be used in cases of primary TR (ex prolapse). In the future, we expect that more experience will accumulate with TTVR, along with innovation, improvement in the design and durability of the devices and a decrease in costs. The number of centers where these procedures are performed is expected to increase.

## 6. Conclusions

In recent years, TTVR has become a viable option for severe secondary TR in patients with high surgical risk and in the absence of severe right ventricular dysfunction, severe pulmonary hypertension or comorbidities that significantly reduce survival. There are four methods of transcatheter repair of which TEER is the most commonly used. The choice of the repair technique depends on the tricuspid valve morphology and on the anatomy of the right heart and great veins. Multimodality imaging is essential in evaluating the morphology and repair options and in intraprocedural guidance. Transcatheter repair reduces TR severity, causes RV reverse remodeling, improves functional status and reduces hospitalizations and mortality.

## Supplementary Materials

The following supporting information can be downloaded at: https://www.mdpi.com/article/10.3390/jcm13226876/s1, Table S1: Trials with tricuspid regurgitation transcatheter repair– baseline patient characteristics; Table S2: Results after transcatheter repair; Table S3: Complications after transcatheter repair of the tricuspid regurgitation. References [43,44] are cited in the supplementary materials.
