# Supplementary material for: Transcatheter Tricuspid Regurgitation Repair—An Overview of Techniques and Eligible Patient Selection
Source: J Clin Med. 2024 Nov 15;13(22):6876. doi: 10.3390/jcm13226876 (PMC11594685; doi:10.3390/jcm13226876)
Supplement: Supplementary file 1 [file jcm-13-06876-s001.zip › jcm-3236081-supplementary.pdf]

Table S1. Trials with tricuspid regurgitation transcatheter repair– baseline patient characteristics

Trials/	device used for repair	no of pts	age	Euro Score 2	TR severity 3+ (%)	NYHA III +IV (%)	EROA (cm2)	vena contracta (mm)	regurgitant volume (ml)	TAPSE (mm)	FAC (%)	tricuspid annulus diameter (mm)	PAP systolic (mmHg)
**TEER**													
TRILUMINATE [9]	TriClip	85	77.8	8.7	92	75	0.65± 0.03	17.3± 0.7	52.2 ± 2.35	14.4± 4.7	36 ±0.85	43.4 ± 0.6	-
TriValve Registry [11]	MitraClip	249	77	6.4	97	95.6	0.7 ± 0.53	9.9 ± 4.1	-	15.8	-	47 ± 7.6	43.6± 16
Ruf [15]	MitraClip	50	80	Euroscore 1 18.25	86	98	-	15.5	-	15.5 ± 3.3	32.65		-
Besler [7]	MitraClip	117	79	6.3	94	97	0.5	9	-	16	40	48.7 ±6.6	-
Orban TTVR/ [23]	MitraClip +PASCAL	111	75	-	100	92	0.61 ± 0.37	10.9± 3.5	-	16.4 ± 0.3	38.7 ±9.9	46.4 ± 7.6	43.4± 14.2
Orban TTMVR [23]	MitraClip	114	77	-	94	98	0.49 ±0.26	9.7 ± 3.3	-	16.1 ± 4.3	35.3 ±8	47 ±6.7	48.7±15.8
Meijerink [16]	MitraClip XTR/ Triclip	21	78	-	100	95	-	14	-	13	-	48	35
Sugiura [19]	MitraClip XTR	22	78	7.5	100	91	0.7	9.6	53.6	18 ± 4.5	-	40.5	-
Braun [20]	MitraClip XTR	18	78	10	94	100	0.64± 0.23	9± 3	64 ± 23	16 ± 5.3	38 ± 12	40	37±12 + RA pressure
Nickenig [18]	MitraClip	64	76	-	88	93	0.9± 0.3	11 ±5	57.2 ± 12.8	16.9 ± 4.3	37.1± 13.1	41.2 ±10.6	42.5±15
Besler [43]	MitraClip	27	79	8.8	74	100	0.51± 0.34	9.2 ± 2.8	50 ± 30	16		51 ±5	44±19
Kodali [17]	Pascal	34	76	5.3	97	79	0.71± 0.33	15± 4.8	47.4 ± 22.5	15.3 ±4.7	38.4± 9	-	-
Sugiura [19]	Pascal	22	79	7.5	100	96	0.74	9.5	51.8	16.5	-	45	-
Fam [13]	Pascal	28	78	6.2	100	100	1.3 ± 0.24	11± 5	57.7± 16	15.7± 3.3	-	47.4	27.8±12.6+ RA pressure
Perlman-1/ [26]	FORMA	18	76	9	94	94	1± 0.6	12 ± 0.3	-	15 ± 5	-	46 ±5	43 ±13
Perlman -US-EFS [26]	FORMA	29	76	8.1	86	84	1.1 ±0.6	16 ± 0.5	-	14 ± 4	-	44 ±7	-
Asmarats [21]	FORMA	19	76	9.2	94.8	93	0.92±0.55	11.8 ± 4	-	15.3 ±4.6	-	46.1 ±5.8	43 ±11

	device used for repair	no of pts	age	Euro Score 2	TR Severity 3+ (%)	NYHA III +IV (%)	EROA (cm2)	vena contracta (mm)	regurgitant volume (ml)	TAPSE (mm)	FAC (%)	tricuspid annulus diameter (mm)	PAP systolic
**Transcatheter valve**													
Hahn [33]	GATE valve	5	84.4	-	100	80	0.71±0.05	-	62.6 ±12.6	11±0.2	35.4 ±7.5	44.6 ±0.3	47.4± 15.6
Fam [31]	Evoque valve	25	76	7.7	100	88	0.85 ±0.2	12.2 ± 2.1	60 ±8	15.6±2.5	37.6± 5.1	44.7 ±7.1	-
TRISCEND [32]	Evoque valve	176	78.7		88	75.4							
**Annuloplasty**													
Nickenig [10]	Cardioband	30	75	4.1	76	83	0.79 ±0.51	12.6 ±0.45	79.4± 29.6			42.2 ±0.5	35.8±10.6
Davidson [27]	Cardioband	30	77.4	-	100	70	0.84 ±0.39	14.8 ±0.48	-	-	41.6 ±5.2	45.2 ±4.6	39± 11
Nickenig [44]	Cardioband	61	78.6	6.8	94	85	0.76 ±0.48	15± 0.56	-	16 ±3	37.7± 6.6	45.5 ±4.5	33±11
Hahn [29]	TriAlign	15	73.2	-		66.7	0.51 ±0.16	13 ±0.3	86 ± 21	16 ±0.4	-	40 ±5	43.6 ±9.3
**Caval valves**													
Lauten [35]	CAVI-Sapien +TricValve	25	73.9	18.2	100	100	-	-	-	13 ±1.8		51 ±6.7	26± 7.7
Loureiro [36]	CAVI-TricValve	35	76	5.8	100	100	0.82	11.4	-	18± 4	47.7 ±8	41 ±9	42.3± 11.3
Wild [37]	CAVI-Tricento	21	76	11	100	95	-	12 ± 5	-	-	46± 12	41 ±7	37 ± 17

EROA- effective regurgitatnt orifice area, TAPSE- tricuspid plane systolic excursion, FAC- fractional area change, PAP- pulmonary artery pressure

Table S2. Results after transcatheter repair

study	device	EROA baseline (cm2)	vena contracta baseline (mm)	EROA decrease (%)	vena contracta decrease (%)	TR severity decrease ≥ 1grade (%)	TR grade 1or 2 at 30 days (%)	tricuspid annulus diameter decrease (%)	NYHA I+ II post-procedural (%)	6MWD increase post procedural (m)	reverse remodeling right heart	30 day mortality (%)	1 year mortality (%)
**TEER trials**													
TRILUMINATE [9]	TriClip	0.65± 0.03	17.3± 0.7	-50	-54	87	63	-	83	31	yes		7.1
TriValve Registry [11]	MitraClip	0.7 ± 0.53	9.9 ± 4.1	-	-	89.2	77	-	69			2.8	20
Ruf [15]	MitraClip	-	15.5	-	-50	98	54	-	56	68	no	0	-
Besler [7]	MitraClip	0.5	9	-60	-44	81	78	-		55		3.4	21 at 184 days
Orban TTVR * [23]	MitraClip +PASCAL	0.61 ± 0.37	10.9± 3.5	-59	-44	82	72	-	67	39		1.8	21
Orban TTMVR* [23]	MitraClip	0.49 ± 0.26	9.7 ± 3.3	-53	-48	82	77	-	69	42		5.2	16
Meijerink [16]	MitraClip XTR/Triclip		14			81	57	-	71			10	-
Sugiura [19]	MitraClip XTR	0.7	9.6			96	68	-	82	33		5	-
Braun [20]	MitraClip XTR	0.64± 0.23	9± 3	-56	-55	100	67	0	67	53		0	37.5
Nickenig [18]	MitraClip	0.9± 0.3	11 ±5	-55	-45	91	86	-19	37	27.6	no	5	-
Besler [43]	MitraClip	0.51± 0.34	9.2 ± 2.8	-43	-35	77	78	-9			yes		22
CLASP TR EF Kodali [17]	Pascal	0.71± 0.33	15± 4.8	-38%	-48%	85	52		89	71	yes	0	-
Sugiura [19]	Pascal	0.74	9.5	-	-	91	50		93	33		5	-
Fam [13]	Pascal	1.3 ± 0.24	11± 5	-	-	85	85	-14	88	95	yes	7.1	-
Perlman-1 [26]	FORMA	1± 0.6	12 ± 0.3	-60	-41	70	50	-6.5	94/79**	69	yes	0	0
Perlman -US-EFS trial [26]	FORMA	1.1 ±0.6	16 ± 0.5	-46	-31	-	-	2.2	72	39	yes	6.89	6.89

Asmarats [21]	FORMA	0.92±0 .55	11.8 ± 4	-16	-28	-	67	-6.5	66	54	yes	0	24 at 32 months
study	device	EROA (cm2) baseli ne	vena contracta (mm) baseline	EROA decrease (%)	Vena contracta decrease (%)	TR severity decrease ≥ 1grade (%)	TR grade 1or 2 at 30 days (%)	Tricuspi d annulus diameter decrease	NYHA I+ II post procedu ral	6MWD imcrease post procedural	Reverse remodeling right heart	30 day mortality (%)	1 year mortality (%)
**Trans catheter valves**													
Hahn [33]	GATE valve	0.71± 0.05				100	100	-		-	yes	20	-
Fam [31]	Evoque valve	0.85 ±0.2	12.2 ± 2.1			96	96	-	76	-	yes	0	-
TRISCEND [32]	Evoque valve					100	97.6		93.3	56.2±11.7	yes	1.7	9.1
**Annuloplasty**													
Nickenig [10]	Cardioband	0.79 ±0.51	12.6 ±0.45	-50	-28	50	67	-10.6	88	60	-	6.7	10
Davidson [24]	Cardioband	0.84 ±0.39	14.8 ±0.48	-35	-39	85	44	-14	75	0	-	0	-
Nickenig [44]	Cardioband	0.76 ±0.48	15± 0.56	-55	-47	78	59	-20	74	-	yes	1.6	-
Hahn [29]	TriAlign	0.51 ±0.16	13 ±0.3	-37	-15	-	-	-	100	43	-	0	-
**Caval valves**													
Lauten [35]	CAVI-Sapien +TricValve	-	-	-	-	-	-	-1.17	52.7	-	-	16	63
Loureira [36]	CAVI-TricValve	0.82	11.4	-4.87	-3.5	13.6	13.6	0	79.4	31	no	2.8	8.5 at 6 months
Wild [37]	CAVI-Tricento	-	12 ± 5	-	-	-	-	-	65	-	yes	0	24

*Orban TTVR- pts with transcatheter tricuspid valve repair/ TTMVR-pts with transcatheter tricuspid and mitral valve repair

Table S3. Complications after tricuspid regurgitation transcatheter repair

technique	device	complications	
TEER	MitraClip<br>TriClip<br>Pascal<br>[7, 10,11, 13, 17, 19, 20,23, 16]	• stroke ±acute myocardial infarction :0- 5.55%<br>• bleeding : 0- 24%<br>• acute kidney injury: 0- 6.3%<br>• infection : 0- 5.4%, endocarditis : 0-0.85%<br>• tamponade :0-9%	• low cardiac output syndrome due to afterload mismatch 2.63-5%<br>• arrhythmia – up to5%<br>• single leaflet device attachment- 1-10%<br>• repeat surgical or transcatheter procedure : 0-14%
	Forma<br>[26, 21]	• stroke± acute myocardial infarction 5.3-6%<br>• bleeding 10.5-12%<br>• acute kidney injury 0- 6%<br>• endocarditis 0- 3.44%<br>• ventricular premature beats 0- 11%<br>• right ventricle perforation 5-11%	• device thrombosis 5.5%<br>• device dislocation 0- 5%<br>• device related cardiac surgery 0- 6%<br>• pulmonary embolism 0- 5%
annuloplasty	Cardioband<br>TriAlign<br>[10, 27, 29, 44]	• stroke± acute myocardial infarction 0-3.3%<br>• bleeding 11-23.3%<br>• acute kidney injury 0- 3.3%<br>• vascular (access site) complications 0-6.6%<br>• tamponade 1.6-3.33%	• coronary complications (kinking, occlusion) 3.33- 10%<br>• AV block 0-3.33%<br>• anchor disengagements (Cardioband) 6.66%<br>• single pledget detachment (TriAlign) 20%
transcatheter valves	Evoque<br>Gate<br>[31,32, 33]	• AV block requiring temporary or permanent pacing 8-25%<br>• fistula between RV and Valsalva sinus<br>• valve thrombosis 4- 25%*<br>• low cardiac output syndrome due to afterload mismatch 0- 8%	• worsening kidney function 4-20%<br>• bleeding 12- 20%
caval valves	TricValve<br>Sapien<br>Tricento<br>[35, 36, 37]	• bleeding 2.85-12%<br>• acute kidney injury-0-19%<br>• stroke-0<br>• systemic inflammatory syndrome (without bacteremia) 0-4.76%<br>• AV block requiring permanent pacing 0- 2.85%	• prosthesis migration in RA 0-8%<br>• shoulder pain 0-28.5%<br>• device thrombosis 0-5.7%<br>• paravalvular leakage 0-9.52%<br>• stent fracture 0-14%<br>• infections- 0-8%
